# Assessment of safety of niraparib maintenance therapy in epithelial ovarian cancer: an updated systematic review and meta-analysis of randomized control trials

**DOI:** 10.1097/MS9.0000000000003186

**Published:** 2025-03-18

**Authors:** Muhammad Saad Khan, Maliha Khalid, Kanza Farhan, Harshika Khaim Chandani, Osaid Ahmed Ansari, Devya Khaim Chandani, Dua Khalid, Usama Memon, Aminath Waafira

**Affiliations:** a Department of Medicine, Jinnah Sindh Medical University, Karachi, Pakistan; b Department of Medicine, Shaheed Mohtarma Benazir Bhutto Medical College, Pakistan; c School of Medicine, The Maldives National University, Malé, Maldives

**Keywords:** epithelial ovarian cancer, hematological side effects, niraparib, safety profile, treatment

## Abstract

**Introduction:**

Patients with epithelial ovarian cancer (EOC) who are treated with niraparib maintenance therapy, experience hematological and gastrointestinal side effects. There is a scarcity of evidence on the safety of niraparib. This study aims to assess the safety profile of niraparib as a maintenance treatment for women with platinum-sensitive EOC.

**Methods:**

PubMed (Medline), EMBASE, and Google Scholar were searched for randomized controlled trials (RCTs) with people suffering from EOC. This study used Review Manager and forest plots for visual display. Random effects models were used for this meta-analysis (RRs and 95% CIs),

**Results:**

The study analyzed 2311 cases from 7/8 RCTS of niraparib-treated patients with high risks of any grade of anemia (RR, 3.51; 95% CI, 2.99 to 4.10, *P* < 0.00001) and thrombocytopenia (RR, 13.28; 95% CI, 10.00 to 17.63, *P* < 0.00001). For grade 3 or 4 adverse effects, significantly higher risk was only noted for thrombocytopenia (RR, 32.80; 95% CI, 10.63 to 101.22, *P* < 0.00001), anemia (RR, 14.45; 95% CI, 6.48 to 32.27, *P* < 0.00001) for niraparib-treated patients. Less treatment-related deaths occurred.

**Conclusion:**

There is a need to emphasize on cautious use, hematological toxicities, and personalized dosage regimens of niraparib for improved patient compliance.

## Introduction

Ovarian cancer (OC) is the leading cause of death among gynecologic cancers^[1]^. The majority of OC cases are diagnosed at an advanced stage, increasing the risk of recurrence and mortality. For patients with distant-stage disease, the estimated 5-year survival rate is approximately 30%^[2]^. The delay in diagnosis is primarily due to the lack of specific symptoms and the limited reliability of current screening methods, such as pelvic ultrasound and CA-125 blood tests.

**HIGHLIGHTS**

- **Ovarian Cancer Challenges**: Advanced ovarian cancer has high mortality due to late diagnosis; treatment now includes Niraparib as a maintenance therapy.
- **Grade ANY Adverse Effects**: Niraparib increased nausea (RR 2.12), vomiting (RR 2.07), constipation (RR 1.99), and hematological issues.
- **Grade 3/4 Adverse Effects**: Niraparib had much higher grade 3/4 anemia (RR 14.45), thrombocytopenia (RR 32.80), and neutropenia (RR 11.07).
- **Overall Impact**: Despite more adverse effects, the study’s low bias ensures reliable results; niraparib caused more GI and hematological issues.
- **Conclusion**: Niraparib shows efficacy in extending progression-free survival but requires careful management of hematological toxicities.

The standard treatment for newly diagnosed advanced epithelial OC (EOC) includes surgical cytoreduction, aimed at removing visible tumor tissue, followed by platinum–taxane combination chemotherapy, typically involving platinum-based agents like cisplatin and taxanes such as paclitaxel. However, recurrence remains a significant challenge, with up to 85% of patients experiencing relapse after completing chemotherapy^[3]^. In cases of recurrent OC, treatment aims to extend survival and improve quality of life. The platinum-free interval plays a crucial role in guiding treatment selection^[4,5]^. For platinum-sensitive recurrences (defined as relapse occurring 6 or more months after initial chemotherapy), the preferred approach remains platinum-based combination therapy. However, resistance to these agents is an eventual concern^[5]^.

The treatment landscape has evolved with the introduction of maintenance therapy following initial chemotherapy or surgery. Maintenance strategies utilize poly (ADP-ribose) polymerase (PARP) inhibitors and antiangiogenic agents, either alone or in combination^[2]^. In 2017, the FDA and EMA approved niraparib, a PARP inhibitor, for maintenance therapy in patients with recurrent EOC who achieved a complete or partial response to platinum-based chemotherapy^[6]^.

PARP enzymes facilitate the repair of single-strand DNA breaks (SSBs). If left unrepaired, these breaks can progress to double-strand DNA breaks (DSBs), leading to cell death^[7]^. PARP inhibitors, such as niraparib, exploit defects in the homologous recombination repair pathway, particularly in tumors harboring BRCA1 and BRCA2 mutations. These mutations impair the repair of DSBs and interstrand cross-links (ICLs), ultimately triggering apoptosis^[8,9]^. By inhibiting PARP, these drugs prevent the repair of SSBs, causing an accumulation of DSBs that BRCA-mutated cells cannot effectively repair – a concept known as synthetic lethality^[10,11]^.

Niraparib offers key advantages by selectively targeting the vulnerabilities of cancer cells while sparing healthy tissue. It has demonstrated a manageable safety and tolerability profile in patients with ovarian OC^[10]^.

The primary objective is to conduct a systematic literature review and meta-analysis, adhering to Preferred Items for Systematic Reviews and Meta-Analysis (PRISMA) guidelines, to assess the safety of niraparib in EOC patients, with a particular focus on adverse effects, dosage modifications, and treatment discontinuations. The goal is to provide insights into its clinical tolerability. However, due to the limited number of available studies, definitive conclusions regarding niraparib’s role in EOC treatment remain uncertain. This systematic review and meta-analysis seeks to identify all clinical trials involving niraparib in EOC patients. By highlighting both its therapeutic benefits and potential risks, this study aims to inform and guide the clinical application of niraparib in EOC management.

## Methodology

### Data sources and search strategy

This meta-analysis was conducted in concordance with the PRISMA guidelines^[11]^ and the work has been reported in line with AMSTAR (Assessing the methodological quality of systematic reviews) guidelines. A comprehensive electronic search on PubMed (Medline), EMBASE, and Google Scholar was conducted, covering the years from inception to June 2024. A search string which included (“Ovarian Neoplasms” OR “Ovarian Cancer” OR “Ovary Neoplasms” OR “Cancer of the Ovary” OR “Ovary Cancers”) AND (niraparib). Two unbiased authors carried out the search without any limitations or consideration. A search was conducted on “clinicaltrials.gov” for relevant published or unpublished clinical trials. Furthermore, we conducted a manual search of the reference lists of the included research, as well as related meta-analyses and review articles, to identify possibly relevant studies.

### Study selection

**Figure 1.**
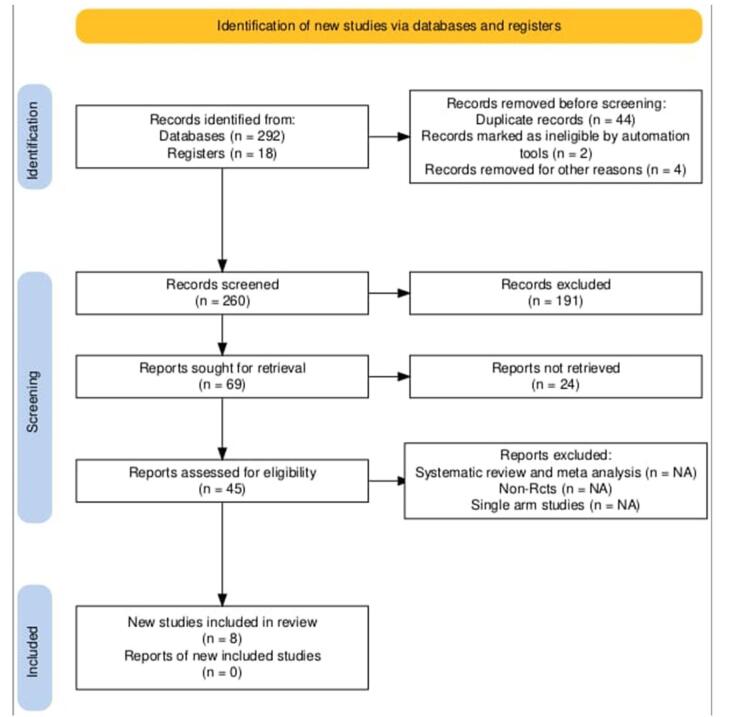
PRISMA flowchart.

This meta-analysis only included randomized controlled trials (RCTs) with a target population of patients diagnosed with platinum-sensitive EOC. Studies comparing niraparib against control (placebo or other treatment) were included. Case reports, review articles, expert opinions, comments, cross-sectional research, and editorials were all removed. The process of choosing our studies is illustrated by the PRISMA chart in (Fig. 1). We found 292 records on PubMed and 18 more items on registries using the right search terms. Once duplicates were found and eliminated, we were left with 260 articles, of which 191 were originally disqualified due to trial design and evaluations. Next, we conducted a thorough analysis of the remaining 69 trials. Among them, 24 were removed as the report was not retrieved, 37 trials were rejected because the selection criteria were not met. Lastly, eight RCTs with 4298 patients were included in our study. Of them, a total of 3565 patients were assigned to either niraparib or control, included in analysis.

### Data extraction and assessment of study quality

**Figure 2.**
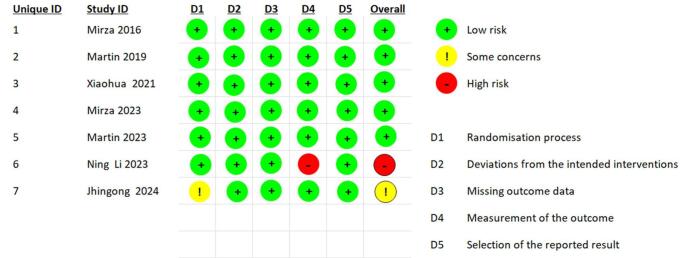
Risk of bias assessment.

Selected articles were first screened based on the abstract and title. After that the remaining articles were screened by going through the full text. A third reviewer was consulted to resolve any disagreements regarding the results. An online Microsoft Excel spreadsheet was created using data from the completed RCTs for the baseline characteristics and outcomes. The baseline parameters are as follows: mean age (SD), starting dose, type of PT treatment, intervention, control, intervention, tumor location, hazard ratio, dose discontinuation, dose interruption, and dose reduction. Following outcomes were included in meta-analysis HRD status, response to therapy, nausea, fatigue, thrombocytopenia, anemia, vomiting, neutropenia, headache, appetite, diarrhea, constipation, abdominal pain, back pain, insomnia, palpitations, nasopharyngitis, cough, dizziness, hypertension, leucopenia. The modified Cochrane Collaboration risk of bias tool 2.0 was utilized to check reliability of RCTs (Fig. 2). Quality assessment was performed using the said tool in all included RCTs by two investigators independently and the results were matched. Any discrepancies were resolved by team consensus.

### Meta-analysis

The meta-analysis was conducted using Review Manager (version 5.4. Copenhagen: Nordic Cochrane Centre, The Cochrane Collaboration, 2014). Forest plots were generated for the results’ visual display. The outcomes were displayed as risk ratios (RR) with a 95% confidence interval using the random effects model. If the reported I2 value was higher than 50%, the outcome was subjected to sensitivity analysis to ascertain the distinct impacts of each study on that specific pooled outcome.

## Results

Seven RCTs^[12-18]^ quoting 3565 patients were randomized, with either niraparib or control maintenance. We excluded one of our PRIMA trial (Pothuri B, *et al* 2024), from analysis, due to not getting quantifiable data from it. Baseline characteristics are summarized in (Table 1) and (Table 2).

**Table 1:** BASELINE CHARACTERISTICS n: number, NR: none reported, gBRCAm (+): germline BRCA mutated positive, gBRCAm (-): germline BRCA mutated negative, HRD (+): homologous recombination deficient positive, Non-HRD (+): non- homologous recombination deficient positive

Mirza, *et al* 2016						González-Martín, *et al* 2019					Wu, *et al* 2020			González-Martín, *et al* 2023	
	BRCA Total no. of patients 203		Non BRCA Total no. of patients 350			HRD (+) Total no. of patients 373			Non-HRD(+) Total no. of patients 249		Total no. of patients 265			Total no. of patients 733	
	Niraparib	Control	Niraparib	Control		Niraparib	Control		Niraparib	Control		Niraparib	Control	Niraparib	Control
Number of patients	138	65	234	116		247	126		240	120		177	88	487	246
Number of patients who discontinued treatment	91	61	188	104		126	83		184	93		101	77	405	217
Number of patients receiving ongoing treatment at data cut off	47	4	46	12		121	42		56	27		76	11	79	27
Median age, years	57	58	63	61		58	58		66	66		53	55	65	65
Primary location tumor															
Ovary	122	53	192	96	201		105	187		96	174		86	NR	NR
Other	16	12	42	19	46		21	53		24	3		2	NR	NR
Response to platinum-based chemotherapy											
Complete	71	33	117	60	185		93	152		79	121		60	213	134
Partial	67	32	117	56	62		33	88		41	56		28	119	65
Number to previous platinum-based regimes											
1	1	0	0	0	NR		NR	NR		NR	NR		NR	NR	NR
2	70	30	155	77	NR		NR	NR		NR	NR		NR	NR	NR
≥3	67	35	79	38	NR		NR	NR		NR	NR		NR	NR	NR

**Table 2:** BASELINE CHARACTERISTICS n: number, NR: none reported, gBRCAm (+): germline BRCA mutated positive, gBRCAm (-): germline BRCA mutated negative, HRD (+): homologous recombination deficient positive, Non-HRD (+): non-homologous recombination deficient positive

Jinhong Chen *et al* 2023			Mansoor R. Mirza *et al* 2023				Ning Li *et al* 2020		Pothuri B *et al* 2024	
	Total no. of patients164		FSD Total no. of patients 475		ISD Total no. of patients 258		Total no. of patients 384		Total no. of patients 733	
	Niraparib	Control	Niraparib	Control	Niraparib	Control	Niraparib	Control	Niraparib	Control
Number of patients	60	104	317	158	170	88	255	129	487	246
Number of patients who discontinued treatment	-	-	204	116	103	59	153	100	-	-
Number of patients receiving ongoing treatment at data cut off	-	-	111	42	66	27	102	29	-	-
Median age, years	57	58	-	-	-	-	53	54	-	-
Primary location tumor										
Ovary	55	101	NR	NR	NR	NR	229	117	NR	NR
Other	5	3	NR	NR	NR	NR	26	4	NR	NR
Response to platinum-based chemotherapy									
Complete	43	69	NR	NR	NR	NR	212	103	NR	NR
Partial	17	32	NR	NR	NR	NR	43	26	NR	NR
Number to previous platinum-based regimens									
<6	44	84	NR	NR	NR	NR	NR	NR	NR	NR
>6	16	20	NR	NR	NR	NR	NR	NR	NR	NR

### Dosage safety analysis

The NOVA study^[12]^ found that 97.5% of patients treated with niraparib and 70.9% of patients treated with control experienced treatment-related side events, with 74.1% experiencing adverse events compared to 22.9% of the control cohort. Nausea (73.6%), thrombocytopenia (61.3%), fatigue (59.4%), and anemia (50.1%) were the most frequently reported any grade adverse events while thrombocytopenia (33.8%), anemia (25.3%), neutropenia (19.6%), and fatigue (8.2%) were the most reported grade 3 or 4 adverse events among niraparib-treated patients. However, dosage reductions of no more than 200 and 100 mg/day were allowed. Patients receiving niraparib reported dosage change (66.5%) and treatment cessation (14.7%) due to treatment-related side events. Patients’ quality of life assessments showed identical results in both groups. Throughout the experiment, no treatment-related deaths were reported.

The PRIMA trial^[13]^ found that 96.3% of patients treated with niraparib experienced treatment-related side events of any grade, with 65.3% experiencing grade 3 or 4 adverse events. The most common any-grade adverse events were thrombocytopenia with decreased platelet count (73.3%), anemia (63.4%), and nausea (57.4%). The most reported grade 3 or 4 adverse events were neutropenia (12.8%), anemia (31%), and thrombocytopenia with decreased platelet count (41.7%). Patients received either a fixed (300 mg/day) or individualized dose of niraparib maintenance or control. Overall, niraparib treatment led to dosage reduction in 70.9% of cases and treatment cessation in 12% of cases. No treatment-related deaths occurred during the experiment.

The NORA trial^[14]^ showed that 99.4% of niraparib patients experienced treatment-related side effects, compared to 87.5% in the control group. Grade 3 or 4 adverse events occurred in 44.6% of niraparib patients versus 11.4% in controls. The most common any-grade side effects included decreased white blood cell count (59.3%), neutropenia (58.8%), decreased platelet count (54.8%), anemia (53.1%), and nausea (53.1%). Grade 3 or 4 events included neutropenia (20.3%), anemia (14.7%), and decreased platelet count (11.3%). Dosage was 300 mg/day initially, with 200 mg/day for patients weighing <77 kg or with platelet counts <150 000/μL. Treatment discontinuation occurred in 4%, and 59.9% required dose modifications. No treatment-related deaths occurred by the trial’s primary cut-off date.

The PRIMA 2 trial^[15]^ reported treatment-emergent adverse events (TEAEs) in 99% of niraparib-treated patients and 93.9% of control-treated patients. Among niraparib patients, common any-grade adverse events included thrombocytopenia (67.1%), anemia (65.1%), nausea (58.3%), and neutropenia (43.2%). Hematologic toxicities mostly occurred in the first month. Initially, all patients received a fixed starting dose (FSD) of 300 mg QD, later adjusted to 200 mg QD for <77 kg or <150 000/μL and 300 mg QD for ≥77 kg or ≥150 000/μL. Dose modification occurred in 66.5%, reduction in 71.7%, discontinuation in 13.8%, and interruption in 80.4%. No treatment-related deaths occurred.

In PRIME^[16]^, Any grade TEAEs were reported niraparib-treated patients (99.2%) and control-treated patients (93.8%).Grade 3 or higher TEAEs were reported in niraparib-treated(57.3%) and thrombocytopenia (52.2%). Whereas the most-reported grade 3 or 4 adverse events were anemia (18.0%), neutropenia (17.3%), leucopenia (6.7%) and hypertension (4.7%). Dose reductions due to TEAEs occurred in (40.4%) niraparib-treated, respectively. A total of 54.4% patients, niraparib (58.9%) and control (41.4%) had disease progression or died. Most TEAEs were well-controlled, which led to low treatment discontinuation rates in niraparib group (6.7%).

In PRIMA 3^[17]^, among niraparib-treated patients, the most reported any grade adverse events included thrombocytopenia (66.3%), anemia (64.3%), nausea (57.4%), neutropenia (42.4%). Initially the starting dose was 300 mg. Subsequently, the protocol was amended so newly enrolled patients received an initial starting dose 200 mg once daily and 300 mg once daily in all other patients. Subgroup A (FSD subgroup [300-mg starting dose] with BW ≥77 kg and PC ≥150 000/μL); subgroup B (ISD subgroup [300-mg starting dose] with BW ≥77 kg and PC ≥150 000/μL); subgroup C (FSD subgroup [300-mg starting dose] with BW <77 kg or PC <150 000/μL); and subgroup D (ISD subgroup [200-mg starting dose] with BW <77 kg . Rates of niraparib dose interruptions because of TEAEs were lower in the ISD subgroup than in the FSD subgroup (71.6% versus. 83.8%), as were rates of dose reductions because of TEAEs (61.5% versus 75.9%). In niraparib-treated patients, incidence of treatment discontinuations because of TEAEs, was similar in both subgroups (FSD, 11.1%; ISD, 13.6%). or PC <150 000/μL).

In Jinghong Chen study^[18]^, among niraparib-treated patients, the most reported any grade adverse events included leucopenia (58.3%), anemia (41.7%), thrombocytopenia (38.3%), nausea (38.3%), and vomiting (21.7%), whereas the most-reported grade 3 or 4 adverse events were anemia (8.3%) and thrombocytopenia (15.0%). A 4.9% experienced dose discontinuation due to adverse effects. Among them, 2.4% discontinued the medication due to grade ≥3 adverse effects. Approximately 39.0% of patients experienced dose reduction, with 7.9% of them related to grade ≥3 adverse effects, such as anemia (3.0%) and thrombocytopenia (1.8%). A 32.9% patients underwent dose interruption due to the most common adverse effects, including anemia (8.5%), leukopenia (7.3%) and thrombocytopenia (7.3%). Few treatment related deaths occur in this trial.

### Meta-analysis of any grade and grade 3 or 4 adverse effects

**Figure 3.**
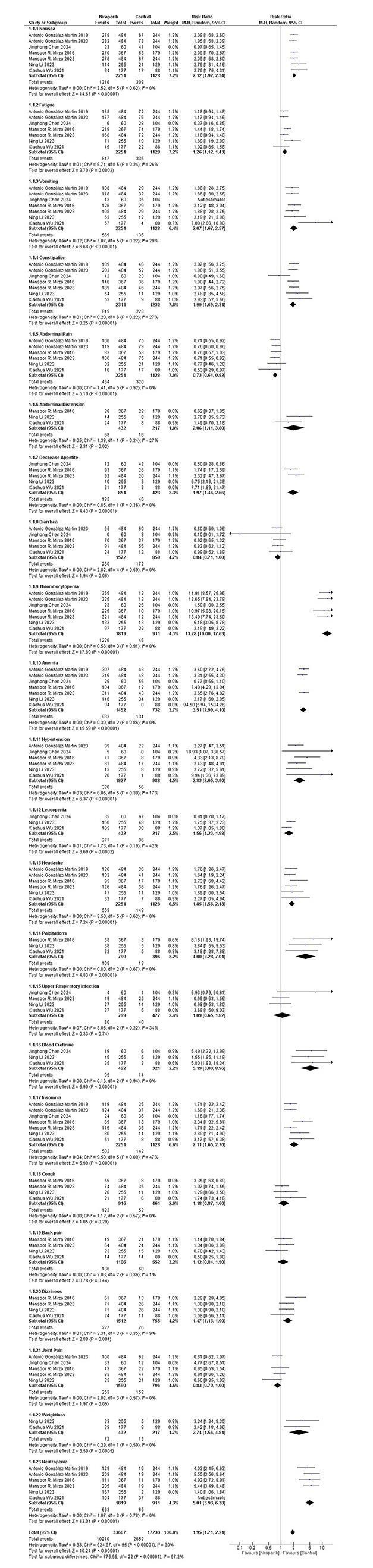
Forest plot of any grade adverse effects.

**Figure 4.**
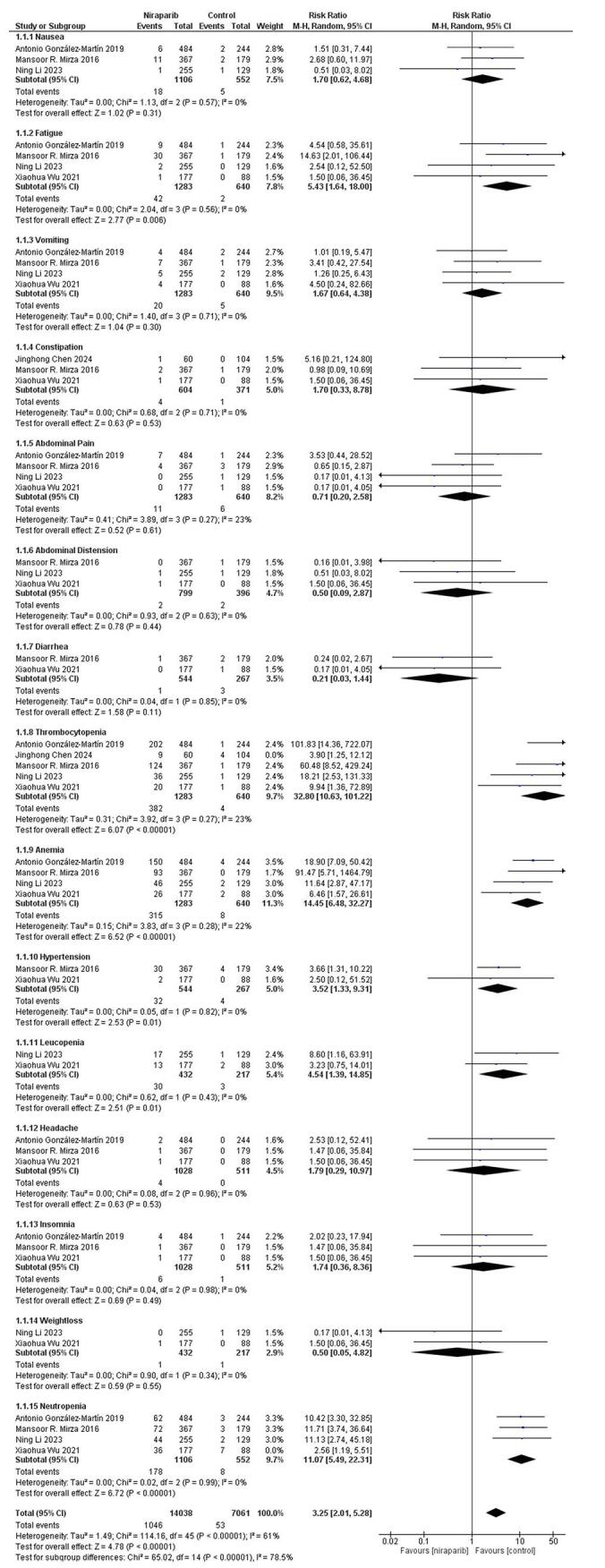
Forest plot of 3 or 4 grade adverse effects.

Adverse effects were recorded in 7/8 studies and the results of our meta-analysis are mentioned in (Fig. 3) and (Fig. 4). In the total 2311 cases of niraparib, of any grade, there were incidences of nausea 1339 of any grade, while from the 1232 cases of control there were 349 cases mentioned (RR, 2.12; 95% CI, 1.92 to 2.34, *P* < 0.00001). As for the rest of the gastrointestinal disturbances, 582 from 2311 patients presented with vomiting episodes, while in the control group only 170 incidents out of 1232 patients were recorded (RR, 2.07; 95% CI, 1.67 to 2.57, *P* < 0.0001). Patients treated with niraparib encountering constipation issues were counted to 845, while those taking control were 223 (RR, 1.99; 95% CI,1.69 to 2.34, *P* < 0.00001). Abdominal pain of any grade was mentioned in 459 patients taking niraparib and in the control group, 283 did experience abdominal pain of any grade (RR, 0.71; 95% CI, 0.59 to 0.86, *P* < 0.00001). As for the hematological adverse effects, 1382 patients treated with niraparib versus 236 control patients presented with anemia (RR, 3.51; 95% CI, 2.99 to 4.10, *P* < 0.00001). Thrombocytopenia of any grade was recorded in 1479 niraparib cases, while in the control group there were only 106 cases (RR, 13.28; 95% CI, 10.00 to 17.63, *P* < 0.00001). Neutropenia was recorded in 924 niraparib-treated patients and in 104 cases in the control group (RR, 5.01; 95% CI, 3.93 to 6.38, *P* < 0.00001). Joint pain was recorded in 286 niraparib cases and in 164 cases in the control group (RR, 0.83; 95% CI, 0.70 to 1.00, *P* = 0.05). 320 patients having hypertension in niraparib group and 56 patients in control group (RR,2.83; 95% CI, 2.05 to 3.90, *P* < 0.00001). Incidents of any grade such as decreased appetite, diarrhea, upper respiratory tract infection, fatigue, headache, insomnia, raised blood creatinine, leucopenia, weight loss, back pain, abdominal distention, palpitations, nasopharyngitis, cough and dizziness were recorded among our trials.

Regarding the grade 3 or 4 adverse effects, we counted 18 patients out of a total 1343 treated with niraparib with grade 3 or 4 nausea, while in the control group, only 5 out of 744 were experiencing the same (RR, 1.70; 95% CI, 0.62 to 4.68, *P* = 0.31). Grade 3 or 4 vomiting was seen in 20 patients taking niraparib and in 5 taking control (RR, 1.67; 95% CI, 0.64 to 4.38, *P* = 0.30). Grade 3 or 4 constipation was recorded in 4 niraparib-treated patients, while in only one case in the control cohort (RR, 1.70; 95% CI, 0.33 to 8.78, *P* = 0.53). Moreover, 11 patients taking niraparib and 6 taking control did experience grade 3 or 4 abdominal pain episodes (RR, 0.71; 95% CI, 0.19 to 2.59, *P* = 0.60). As for the grade 3 or 4 hematological adverse effects, anemia was noted in 320 patients taking niraparib, while the control group counted only 18 patients (RR, 14.45; 95% CI, 6.48 to 32.27, *P* < 0.00001). Thrombocytopenia-recorded episodes were 391 in the niraparib group and 8 in the control cohort (RR, 32.80; 95% CI, 10.63 to 101.22, *P* < 0.00001). Neutropenia grade 3 or 4 was seen in 214 patients in the niraparib group, while 15 patients presented with severe neutropenia among the control group (RR, 11.07; 95% CI, 5.49 to 22.31, *P* < 0.00001). The rest of grade 3 or 4 adverse effects that were noted in our studies included fatigue (RR, 5.43; 95% CI, 1.64 to 18.00, *P* = 0.006), headache (RR, 1.79; 95% CI, 0.29 to 10.97, *P* = 0.53), abdominal distension (RR, 0.50; 95% CI, 0.09 to 2.87, *P* = 0.44), insomnia (RR,1.74; 95% CI, 0.36 to 8.36, *P* = 0.49), diarrhea, leucopenia, hypertension, weight loss and joint pain.

## Discussion

This updated meta-analysis included data from seven RCTs^[12-18]^ with a total of 4298 patients, the previous meta-analysis only included three available phase III RCTs comparing niraparib versus placebo in patients with platinum sensitive high-risk EOC^[19]^. Specifically, niraparib was associated with increased rates of nausea, vomiting, constipation, anemia, thrombocytopenia, and neutropenia across the trials. The objective of this research is to conduct a thorough evaluation of niraparib’s safety as a maintenance treatment, offering novel perspectives on its clinical characteristics. Quantifying the relative risks (RR) of any-grade and severe (grade 3 or 4) adverse events reported across pertinent clinical trials is the goal of this meta-analysis. Our study aims to confirm and extend previous findings regarding adverse event profiles associated with PARP inhibitors, such as rucaparib^[20]^ and olaparib^[21,22]^, which have demonstrated significant benefits in the treatment of OC in patients with a BRCA mutation. Niraparib treatment resulted in a considerable increase in a variety of adverse events when compared to controls, with notable differences in both any-grade and grade 3 or 4 occurrences.

Niraparib was consistently associated with greater rates of hematologic adverse events, including anemia, thrombocytopenia, and neutropenia. Previously, it was discovered that thrombocytopenia was the most common side event that led patients to dosage reduction^[23]^. A recent meta-analysis by Ruiz-Schutz, *et al*^[24]^ found that olaparib therapy was associated with an increased risk of fatigue and anemia when compared to other therapies, which is consistent with our findings. These occurrences were mostly of any grade, but were substantially more common in niraparib as compared to controls. This shows a strong effect of niraparib on hematological parameters, which should be closely monitored during treatment.

Nausea, vomiting, and constipation were notably more common in patients receiving niraparib. While these symptoms were generally manageable, their higher prevalence indicates a need for proactive management strategies to improve patient tolerance and adherence to treatment^[12,13]^.The same findings in quality of life assessments between the niraparib and control groups indicate that, despite greater adverse event rates, these did not significantly impair overall patient-reported outcomes. This was demonstrated even further by the ENGOT-OV16/NOVA study. Patients with recurrent OC have demonstrated significantly improved progression-free survival (PFS) after receiving niraparib maintenance medication. When compared to control, results indicate that women with recurrent OC who respond to platinum treatment and are given niraparib as a maintenance drug are able to preserve their quality of life throughout their treatment^[25]^. However, tailored measures to reduce side effects may improve patient comfort and treatment compliance as per the trials.

Our meta-analysis shows that niraparib maintenance medication significantly raises the likelihood of grade 3 or 4 fatigue, anemia, thrombocytopenia, and neutropenia. Although grade 3 or 4 gastrointestinal side effects, such as nausea, vomiting, and constipation, were common but in our previous meta-analysis they were not statistically significant. Notably, whereas serious adverse events were less common overall, individuals taking niraparib had a 35-fold increased risk of having grade 3 or 4 thrombocytopenia. These data highlight the significance of careful adverse event management in patients receiving niraparib.

The lack of treatment-related mortality across the trials highlights its overall safety profile, despite the greater prevalence of adverse events with niraparib. This suggests that it is a potential maintenance therapy option in adequately selected patients. The prevalence of adverse event-related dose reductions and interruptions underscores the significance of adaptable dosing techniques in clinical settings^[26-28]^. Tailored dosage according to baseline parameters (body mass, platelet count, etc.) has demonstrated potential in lowering severe toxicities without compromising therapeutic efficacy.

All included clinical trials are highly reliable. However, there are some controversies that need to be addressed. To increase the robustness of the meta-analysis results, sensitivity analyses were carried out to make sure that study heterogeneity did not exceed 50%. Despite differences in trial techniques and patient demographics, this strategy aids in more reliably evaluating the pooled results. The exclusion of particular studies or data points due to a lack of quantifiable data, eg. Pothuri B, *et al* 2024^[29]^ demonstrates potential limitations in full data availability across all trials. The study of Pothuri B, *et al* was excluded as it included outcomes measured in cycles which were months of follow up, this made it difficult to obtain desired outcome of adverse effects. Additionally, the grade was also not mentioned. Future research should strive for more consistent reporting in order to do full meta-analytical assessments. Studies like Jinghong Chen, *et al*^[18]^ with 0.00% weightage in outcomes like fatigue, joint pain, leukopenia and vomiting were not included in statistical analysis. The main cause of this study’s heterogeneity is probably the variation in patient groups and follow-up techniques. Patient outcomes can be greatly impacted by variations in variables such as age, BRCA gene status, FIGO staging, histology, neoadjuvant chemotherapy (NACT), residual disease following surgery, and chemotherapy response. Additionally, inconsistent reporting of adverse events and survival statistics may result from the use of various follow-up techniques, such as online community groups, in-person visits, and telephone interviews. These differences in characteristics of patients and data gathering techniques add to the meta-analysis’s found heterogeneity. Some other studies like Ning Li, *et al*^[16]^ and Xiaohua, *et al*^[14]^, in outcomes like neutropenia were also not included in statistical analysis .The study’s heterogeneity is due to a variety of patient factors, such as different cancer types and treatment responses, as well as stratification by gBRCA and HRD status. Furthermore, tailored starting doses of niraparib based on body weight and platelet count add to outcome variability.

In addition to the trials that are included, there are a few other noteworthy interim studies whose results support the conclusions drawn from our research. A multicenter retrospective real-world study conducted in China with 199 patients suffering from advanced OC evaluated the safety and PFS of niraparib therapy. Age under 65, BRCA mutations, and R0 status following surgery were found to be significant variables for longer PFS using LASSO regression. A prediction model that exhibited great sensitivity and accuracy was developed. Anemia and a decline in blood cell counts were among the main side effects, supporting results of our meta-analysis. Compared to patients on 100 mg of niraparib, patients on 200 mg showed greater 18-month PFS rates. According to the study’s findings, Chinese OC patients can safely and effectively take niraparib at a dose of 200 mg^[27]^. Furthermore a health technology assessment also proved that when compared to no maintenance therapy, niraparib enhanced the OC of patients with newly diagnosed (advanced) or recurrent high-grade serous or endometrioid OC tumors with HRD or HRP (GRADE: High)^[30]^.A single center retrospective study with a primary endpoint was the 24-month PFS rate also proves the efficacy with no safety signal except skin pigmentation^[31]^.The QUADRA study assessed the efficacy and safety of single-agent niraparib in late-line treatment in a cohort that is representative of real-world patients^[32]^. Its post-hoc analysis found that patients with lower baseline bodyweight and platelet count had significantly greater incidences of severe thrombocytopenia, neutropenia, and anemia than those with higher baseline values^[33]^. Importantly, response rates, clinical benefit, and disease control rates were comparable across individuals treated with 300 mg and 200 mg daily dosages of niraparib. This implies that a lower dose of 200 mg/day may lessen toxicity concerns while maintaining PFS, underlining the possibility for dose optimization in clinical practice.

As we continue to investigate the potential of PARP inhibitors in clinical settings, the incorporation of these drugs into initial therapy paradigms remains an important focus of research. Furthermore, continuing research is looking into the synergistic effects of PARP inhibitors when taken with immune checkpoint inhibitors (ICIs). These combinations aim to take advantage of the enhanced genomic instability and immunological activation caused by PARP inhibition, which may improve treatment efficacy against a variety of cancers^[34]^.As data evolves, it is critical for physicians and stakeholders to stay current of these advances and ensure that treatment guidelines are revised properly to deliver the best possible outcomes for patients^[35]^ .

## Strengths and limitations of study

Among the many advantages of our study were the following: (1) Strict eligibility criteria ensure that only studies that satisfied predetermined inclusion and exclusion criteria were taken into consideration, which enhances the quality and relevance of the data included in the meta-analysis. (2) The dependability of our results was increased because every RCT that we included in our meta-analysis was a high-quality, multicenter study. (3) The minimal heterogeneity in the majority of the outcomes strengthens the internal validity of our findings. (4) Our meta-analysis is a noteworthy contribution that provides a current perspective on the use of niraparib in patients with high-risk, platinum-sensitive EOC. (5) A more nuanced knowledge of the medications’ safety is made possible by the analysis based on dosages and various outcomes, which offers valuable information to guide clinical decision-making.

It is imperative that we acknowledge certain limitations in our study in order to maintain transparency. There was some variation in our results. The fact that only RCTs were included may limit how broadly applicable the results are. Second, some study did not publish its findings during the maintenance phase, which could have had an impact on the overall findings. Furthermore, owing to heterogeneity and unquantifiable data, certain studies were eliminated in certain results. Notwithstanding these limitations, the research provides valuable insights that warrant investigation in other studies to expand the corpus of data.

## Conclusion

This meta-analysis underscores the safety of niraparib as a maintenance medication for patients with platinum-sensitive EOC. However, healthcare practitioners must continue to monitor and manage side effects, particularly hematological toxicities such as anemia, thrombocytopenia, and neutropenia. This research highlights the significance of personalized dosage protocols, which have demonstrated a reduction in the occurrence of serious adverse events and an enhancement in patient adherence. Note that only data from RCTs involving patients with OC are included in this analysis. As a result, without additional data, the results cannot be extrapolated to other forms of cancer. Future investigations should concentrate on long-term follow-up studies to evaluate the safety and efficacy of niraparib over time, as well as its possible uses in other cancers. In order to improve patient care standards and treatment plans in oncology practice, updated meta-analyses that take into account new data will be crucial. Updated meta-analyses that include new data will be crucial for enhancing treatment options and improving patient care standards in oncology.

## Data Availability

Not applicable.

## Research registration unique identifying number (UIN)

- Name of the registry: PROSPERO
- Unique identifying number or registration ID: CRD42024596664
- Hyperlink to your specific registration (must be publicly accessible and will be checked): CRD42024596664
